# Genetics and Genomics Approaches for Diagnosis and Research Into Hereditary Anemias

**DOI:** 10.3389/fphys.2020.613559

**Published:** 2020-12-22

**Authors:** Roberta Russo, Roberta Marra, Barbara Eleni Rosato, Achille Iolascon, Immacolata Andolfo

**Affiliations:** ^1^Department of Molecular Medicine and Medical Biotechnologies, University of Naples Federico II, Naples, Italy; ^2^CEINGE Biotecnologie Avanzate, Naples, Italy

**Keywords:** hereditary anemias, next generation sequencing, differential diagnosis, chromatin 3D architecture, genetic test

## Abstract

The hereditary anemias are a relatively heterogeneous set of disorders that can show wide clinical and genetic heterogeneity, which often hampers correct clinical diagnosis. The classical diagnostic workflow for these conditions generally used to start with analysis of the family and personal histories, followed by biochemical and morphological evaluations, and ending with genetic testing. However, the diagnostic framework has changed more recently, and genetic testing is now a suitable approach for differential diagnosis of these patients. There are several approaches to this genetic testing, the choice of which depends on phenotyping, genetic heterogeneity, and gene size. For patients who show complete phenotyping, single-gene testing remains recommended. However, genetic analysis now includes next-generation sequencing, which is generally based on custom-designed targeting panels and whole-exome sequencing. The use of next-generation sequencing also allows the identification of new causative genes, and of polygenic conditions and genetic factors that modify disease severity of hereditary anemias. In the research field, whole-genome sequencing is useful for the identification of non-coding causative mutations, which might account for the disruption of transcriptional factor occupancy sites and *cis-*regulatory elements. Moreover, advances in high-throughput sequencing techniques have now resulted in the identification of genome-wide profiling of the chromatin structures known as the topologically associating domains. These represent a recurrent disease mechanism that exposes genes to inappropriate regulatory elements, causing errors in gene expression. This review focuses on the challenges of diagnosis and research into hereditary anemias, with indications of both the advantages and disadvantages. Finally, we consider the future perspectives for the use of next-generation sequencing technologies in this era of precision medicine.

## Introduction

The hereditary anemias (HAs) represent a particularly heterogeneous group of disorders with rare to low frequency that are characterized by complex genotype–phenotype correlations that remain to be explained. It has only been in recent years that major advances have been made in our understanding of the genetic basis and the pathophysiology of HAs. Indeed, more than 70 genes involved in red blood cell (RBC) physiology have been identified as causative of HAs to date.

Based on clinical manifestations and morphological RBC alterations, HAs can be broadly classified into four different subtypes: (i) disorders of hemoglobin (Hb) synthesis, such as thalassemia and hemoglobinopathies; (ii) hyporegenerative anemias, such as Diamond-Blackfan anemia and congenital dyserythropoietic anemias; (iii) RBC membrane defects that are due to either altered structural organization of their membranes, such as hereditary spherocytosis and hereditary elliptocytosis, or to alterations to membrane transport functions, such as hereditary stomatocytosis; and (iv) non-spherocytic hemolytic anemias due to RBC enzyme defects.

In the last few years, much time and effort has been spent on the identification of the genes and mutations that underlie HAs. The rapid evolution in the diagnostic and research technologies involved now needs to be taken into account. The use of such new techniques has changed the ways in which both diagnosis and research are carried out.

This review focuses on the past, present, and future genetics and genomics approaches for establishment of correct differential diagnosis among these conditions, and for research into new causative/modifier genes and new pathogenetic mechanisms.

## Classification of the Hereditary Anemias

Initially, we will briefly describe the main hallmarks, both clinical and molecular, of the various subtype of HAs. Specific guidance on the pathway to establish correct diagnosis for these conditions has been extensively reviewed elsewhere (Da Costa et al., 2013; Andolfo et al., 2016, 2018b; Gambale et al., 2016; Taher et al., 2018; Bianchi et al., 2019; Grace et al., 2019a; Iolascon et al., 2019, 2020), and so this will not be discussed in any detail in the present review.

### Disorders of Hemoglobin Synthesis

The globin disorders can generally be classified as quantitative (e.g., thalassemias) and qualitative (e.g., hemoglobinopathies) defects that lead to hemolysis, and that are defined according to the globin chains. Thalassemia has been shown to be one of the most common genetic disorders worldwide, and it arises as a result of mutations in the α-globin or β-globin genes (Taher et al., 2018). The mutations associated with thalassemia now number over 1,530, and these range from single-nucleotide variations to large genome rearrangements (Higgs et al., 2012; Zhao et al., 2020). α-Thalassemia arises from deletions in the *HBA* genes in ∼95% of patients, while the remaining cases arise from point mutations. In contrast, some 95% of β-thalassemias arise either from *HBB* gene point mutations that result in disruption of RNA transcription, processing or stability, or from nonsense mutations that result in the production of abnormal proteins or in nonsense-mediated decay of RNA. Many of the aspects of β-thalassemia pathophysiology are now explained by excess production of α-globin chains, with the result that they can precipitate in RBC precursors (resulting in ineffective erythropoiesis) and mature RBCs (resulting in hemolysis). Structural variants of the globin genes are associated with sickle-cell anemia, hemolysis caused by the unstable Hb, the altered oxygen affinity of Hb, and Hb where the ferrous (Fe^2+^) state of the iron cannot be maintained (Rees et al., 1999; Sabath, 2017). The use of various electrophoretic techniques to separate the various Hb states has become the mainstay for the diagnosis of these Hb disorders.

### Hypo-Regenerative and Hypo-Productive Anemias

These represent a heterogeneous group of disorders that have effects on the normal differentiation–proliferation pathways at the different steps through the erythroid lineage, and that mainly result in monolinear cytopenia.

Diamond-Blackfan anemia (DBA) is defined by macrocytic moderate or severe anemia that can occur in association with hyporegenerative bone marrow and with reticulocytopenia. Almost half of these patients have physical abnormalities, with 50% showing craniofacial anomalies, and 38% showing defects of the upper limb and hand, which mainly include the thumb. This disease shows mutations in 20 genes for ribosomal proteins, of a total of 80 genes that encode the complete ribosome. For six of these 20 causative genes (i.e., *RPS19*, *RPS24*, *RPS26*, *RPL5*, *RPL11*, *RPL35a*), the mutations and deletions comprise 70% of all DBA patients (Da Costa et al., 2018). There is strong evidence that suggests that erythroid blockage occurs between the BFU-e and CFU-e erythroid development stages, although the exact stage remains to be fully defined (Ohene-Abuakwa et al., 2005).

Congenital dyserythropoietic anemias (CDAs) are a large group of hypo-productive anemias that have been classified into five subtypes: types I, II and III CDAs; CDAs related to transcription factors; and variant CDAs. The most common forms are CDA types I and II (Heimpel et al., 2010), which are caused by bi-allelic mutations in the *CDAN1/CDIN1* genes for CDAI (Dgany et al., 2002; Babbs et al., 2013), and in the *SEC23B* gene for CDAII (Bianchi et al., 2009; Schwarz et al., 2009). For many years, the main diagnostic features of these CDAs were morphological abnormalities of bone marrow (e.g., erythroid hyperplasia with binuclearity) or late erythroblast multinuclearity. However, these particular features of CDAs are not always specific; in particular, they are also seen for other acquired conditions that involve erythropoietic stress, including iron deficiency and preterm birth (Iolascon et al., 2020).

### Red Blood Cell Membrane Disorders

RBC membrane disorders consist of hemolytic anemias, which can show wide differences in their clinical, morphological, laboratory, and molecular aspects. The main effects that these genetic alterations have relate to lowered RBC deformability and shortened RBC survival (Iolascon et al., 2019). Among these, there are hereditary spherocytosis, hereditary elliptocytosis, hereditary pyropoikilocytosis, and Southeast Asian ovalocytosis, which are caused by altered membrane structural organization. Hereditary spherocytosis is the most frequent form, and it is characterized by phenotypic, locus, and allelic heterogeneity. Indeed, mutations in five genes that encode proteins that are involved in interactions between the cytoskeleton and the RBC phospholipid bilayer are associated with these conditions: *ANK1*, *SPTA1*, *SPTB*, *SLC4A1*, and *EPB42* (Iolascon et al., 2019). The clinical manifestations can range from symptom-free carriers to patients with severe hemolysis, jaundice, reticulocytosis, splenomegaly, and cholelithiasis (Da Costa et al., 2013; Andolfo et al., 2016). Conversely, hereditary RBC membrane disorders can arise from genetic defects of RBC transport proteins, which leads to abnormal cation permeability, and the consequent changes in RBC hydration. Hereditary stomatocytosis represents this wide spectrum of diseases, among which the most frequent form is dehydrated hereditary stomatocytosis (DHS). Cation leaks cause dysregulation of cellular volume, which leads in turn to morphological abnormalities of RBCs, with stomatocytes seen for peripheral blood smears, and the consequent leftward shift in the osmolarity curve seen by ektacytometry for patients with mutation of the *PIEZO1* gene (Andolfo et al., 2018a). As generally seen for all hemolytic conditions, the key symptoms include pallor, fatigue, jaundice, gallstones, and splenomegaly. DHS is an autosomal dominant disease that is caused by gain-of-function mutations in *PIEZO1* (DHS type I) and in *KCNN4*, also known as ‘Gardos channelopathy’ due to its peculiar characteristics, which include lack of clear signs of RBC dehydration and normal ektacytometric curve (Zarychanski et al., 2012; Andolfo et al., 2013a, 2018b; Rapetti-Mauss et al., 2015; Picard et al., 2019).

### Non-sperocytic Hemolytic Anemias Due to RBC Enzyme Defects

Mature RBCs rely exclusively on glycolysis for their energy production. Indeed, mutations to almost any of the glycolytic enzymes can result in hemolytic anemias (van Wijk and van Solinge, 2005). The most common human defect is deficiency of glucose-6-phosphate dehydrogenase (G6PD). About 140 mutations in the *G6PD* gene that have X-linked inheritance can cause G6PD functional variants, with many biochemical and clinical phenotypes seen. Of the clinical phenotypes, the main ones are neonatal jaundice and acute hemolytic anemia; this latter is generally triggered by exogenous agents (Luzzatto et al., 2016). Similarly, pyruvate kinase deficiency is generally the cause of chronic non-spherocytic hemolytic anemia, which is an autosomal recessive disease. More than 250 mutations in the *PKLR* gene that encodes the liver and RBC pyruvate kinase isoforms are known to be causative of this condition. Pyruvate kinase deficiency is characterized by a highly variable clinical spectrum (Grace et al., 2018), with patients with two non-missense mutations more severely affected (Bianchi et al., 2020). Furthermore, there are other more rare enzyme defects that are associated with HAs that involve the following: adenylate kinase, aldolase, phosphofructokinase, phosphoglycerate kinase, glucose phosphate isomerase, glutathione reductase, glutathione synthetase, hexokinase, pyrimidine-50-nucleotidase, and triosephosphate isomerase (Koralkova et al., 2014).

## Past, Current, and Future of Molecular Testing for Diagnosis of Hereditary Anemias

Here, we will briefly describe the past, current, and future methodologies for genetic testing of HAs, and highlight the strengths and limitations of these different approaches.

### First-Generation Sequencing: Sanger Method

The conventional workflow for diagnosis of these conditions started as first line of investigation with positive familial history, complete blood count, and peripheral blood smear. Then specialized biochemical tests, and eventually bone-marrow aspirate, were required. Finally, genetic testing by Sanger sequencing served as the confirmatory test. Very often, no mutations in the candidate gene were identified by this approach for the genetic heterogeneity of the conditions, which led to confusing or lacking molecular diagnoses.

**FIGURE 1:**
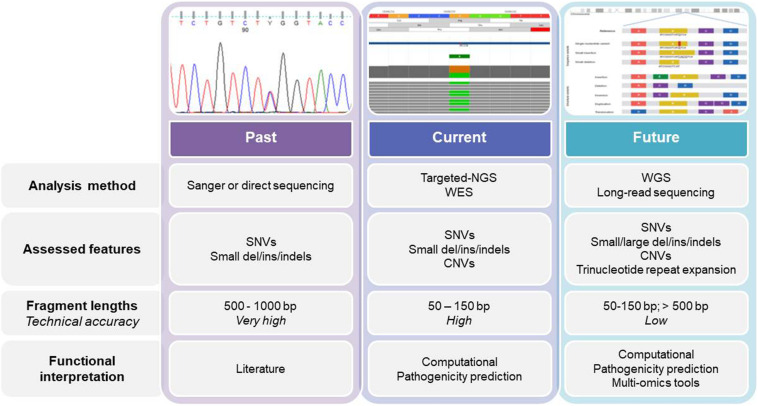
The past, present, and future of genetics and genomics technologies for the diagnosis of and research into hereditary anemias. The diagram shows the current analysis methods for DNA sequencing, while also highlighting the main differences among them. WES, whole-exome sequencing; WGS, whole-genome sequencing; SNV, single nucleotide variant; CNV, copy number variation.

Currently, genetic testing is used early in the diagnostic workflow of HAs, which removes the need for some of the specialized tests, such as bone marrow biopsies (Roy and Babbs, 2019), especially when the clinical data for the patients are not informative, or when the patient is transfusion dependent. Sanger sequencing was also our starting point, while we now use second-generation sequencing (i.e., NGS), which allows us to move from a monogenic approach to an oligo/multigenic approach (Figure 1). For many years, monogenic approaches were used for diagnosis and identification of new causative genes of HAs. For mapping to define the causative gene, there was the need to find families with confirmed Mendelian inheritance that preferably involves multiple generations. The use of linkage approaches associated with the functional mapping of the candidate genes was used to successfully identify the mutations responsible for several HAs, such as in the *SEC23B* gene for CDAII, or in *CDAN1* for CDAI, and in *ABCB6* for familial pseudohyperkalemia (Dgany et al., 2002; Schwarz et al., 2009; Andolfo et al., 2013b).

A critical aspect here is that this traditional approach still has great value, mainly in the diagnostic field. Indeed, the testing of single genes is preferred when: (i) the patient’s clinical features are typical of a specific HA; (ii) an association has been established between the disorder and the specific gene; and (iii) there is complete phenotyping (i.e., at the clinical, biochemical, and morphological levels). For example, for G6PD deficiency, starting with single-gene analysis is usually still preferred, because in most of the cases an assured clinical diagnosis can be obtained, and *G6PD* is the only causative gene of this condition. Such single-gene tests show high clinical sensitivity because the phenotype and further findings indicate a single disorder associated with a single gene. Furthermore, one particular interpretive advantage of this single-gene approach is that there is minimal likelihood of uncovering multiple confounding variants that have unknown clinical significance. On the other hand, for many other HAs, the clinical variability and the heterogeneity of the genetic locus are significant enough such that a custom targeting panel and/or whole-exome sequencing (WES) are the most appropriate for efficient and timely molecular diagnosis.

### Second-Generation Sequencing: NGS Short Reads

Next-generation sequencing has revolutionized the framework of HA diagnosis (Figure 1). Although WES or WGS can provide more information (e.g., identification of non-coding causative mutations, or new causative genes), a drawback remains in that the overall complexity of the data analysis includes a number of variants of unknown significance. The functional tests are therefore crucial to assess the pathogenicity of new variants detected by NGS. Moreover, further problems can arise from the need for adequate coverage of the genes when there are copy number variants and GC−rich regions with low−complexity (Rets et al., 2019). Currently, a targeted-NGS approach is preferred in the diagnostic work-up for these disorders (Agarwal et al., 2016; Del Orbe Barreto et al., 2016; Niss et al., 2016; Roy et al., 2016; Russo et al., 2018; Choi et al., 2019; Kedar et al., 2019; Svidnicki et al., 2020). Custom panels for HAs include variable numbers of genes (e.g., 50–200) that can provide diagnostic yields of 38–87%, which depends on how many and what types of genes are included, and on the depth of the phenotypic assessment (Table 1). The main limitation of the targeted NGS approach is the need for continuous updating of the gene list for each panel, to include all recently identified causative genes. Indeed, the targeted NGS approach does not allow for the identification of new genes, by design. Moreover, the cost of WES is now similar to the cost of the large panels that are required to cover the full gamut of potential gene mutations that underpin HAs. Therefore, in the near future, WES with the use of managed variant lists that contain known pathogenic variants will become the most appropriate diagnostic approach for investigation of HAs (excluding the hemoglobinopathies, which require more specialized tests).

**TABLE 1:** Case series of hereditary anemias diagnosed by targeted next-generation sequencing.

Hereditary anemia subtypes	Families (n)	Patients (n)	Genes (n)	Diagnostic yield (%)^‡^	References
CDA; DBA; sideroblastic anemia; RBC enzymatic defects	57	57	33	38.6	Roy et al., 2016
RBC membrane defects; CDA; RBC enzymatic defects	10	10	40	100^§^	Del Orbe Barreto et al., 2016
RBC membrane defects; RBC enzymatic defects	15	15	28	86.7	Agarwal et al., 2016
RBC membrane defects	13	15	12	100^§^	Niss et al., 2016
RBC membrane defects; CDA; DBA; RBC enzymatic defects	62	74	71	64.9	Russo et al., 2018
RBC membrane defects; CDA; DBA; RBC enzymatic defects	21	21	76	61.9	Shefer Averbuch et al., 2018
BMFS; CDA; RBC enzymatic defects; hematological malignancies	21	21	76	81.0	Kedar et al., 2019
RBC membrane defects; RBC enzymatic defects; hereditary anemia modifiers	59	59	43	84.7	Choi et al., 2019
CDA; RBC membrane defects; RBC enzymatic defects	26	36	35	72.2	Svidnicki et al., 2020

*^‡^Ratio of number of diagnosed patients/numbers of tested patients. ^§^These studies reported only the number of diagnosed patients. CDA, congenital dyserythropoietic anemia; DBA, Diamond-Blackfan anemia; RBC, red blood cell; BMFS, bone marrow failure syndrome.*

The exome is believed to represent 1–2% of the genome; however, it also contains 85% of the mutations that are known to cause disease. WES has been reported to have a diagnostic yield of ∼30–50% when used in clinical diagnostics, which depends on detailed phenotyping (Alkan et al., 2011; Sankaran et al., 2012; Rehm et al., 2013; Hamada et al., 2018). Thus, ∼30–50% of cases will remain undiagnosed following NGS testing. This arises mainly because of incomplete phenotyping of the patients, which reduces the specificity of the data, as supported by a report of the numerous variants of unknown significance (Trujillano et al., 2017). Contrary to the targeted-NGS and WES approaches, no estimate of the diagnostic rate of WGS for HAs has been established yet. In a prospective study with 100 patients referred to a pediatric genetics service, genetic variants that met the clinical diagnostic criteria were identified by WGS in 34% of cases (Stavropoulos et al., 2016).

Next-generation sequencing has many advantageous aspects, which include the low cost and the high speed and yield. Conversely, there are some intrinsic limitations of NGS that can have significant impact on accuracy of these analyses. Considering these as bottlenecks, the most noticeable drawback is the short read length. Short read length has been shown to limit precision in a number of biological studies, in particular for genome assembly and transcriptome analyses. Here, NGS short reads are liable for unresolved complexities that can arise from heterozygosity, GC-rich regions, transposable elements, tandem repeats, and repetitive regions (10 kb–10 Mb or more) interspersed in the genome (Alkan et al., 2011). The use of NGS to sequence polymorphic tandem repeats in the genome can be severely impaired by read length (Mousavi et al., 2019).

Considering the analysis of complex chromosomal rearrangements and structural variants, the analytical approaches of comparative genomic hybridization and multiplex ligation-dependent probe amplification are still widely used for detection of copy-number changes (Minervini et al., 2020). For instance, these techniques are crucial for β-thalassemia diagnosis. Indeed, most β-thalassemia alleles (∼90%) are point mutations, and are thus easily identified through Sanger sequencing or other dedicated methods. As the remaining 10% of the alleles are deletions, these are detectable by comparative genomic hybridization arrays or multiplex ligation-dependent probe amplification (Joly et al., 2014). It is important, however, that the application of all of these techniques should not be considered as mutually exclusive or as consecutive, but rather as synergistic. Indeed, several cases of apparent recessive inheritance due to uniparental isodisomy in probands with homozygous recessive mutations where only one parent is heterozygous for the same variant have been described among patients with HAs (Bogardus et al., 2014; Andolfo et al., 2020).

### Third-Generation Sequencing: NGS Long Reads

The use of NGS with improved read lengths represents a milestone in the study of the genetics of HAs. This so-called third-generation sequencing now has two advantages that are crucial: single molecules can be sequenced without the need for PCR amplification, which thus avoids any PCR bias; and secondly, this also generates read lengths that are longer (Furst et al., 2020). The two main systems that are based on this third-generation NGS are from Pacific Biosystems^1^ and from Oxford Nanopore Technologies^2^. These both have the advantage of long read lengths, although they also share the disadvantage of high randomly distributed error rates (∼5–20%). Indeed, despite their advantages, such methods that provide long reads can also show high error rates, thus reducing the accuracy of this genome sequencing (Minervini et al., 2020). Recently, nanopore sequencing has been used in a number of fields, such as genomics, epigenomics, and transcriptomics. The third-generation NGS approach overcomes the problems of (i) sequencing of tandem repeats; (ii) detection of complex chromosomal rearrangements and structural variants; (iii) RNA sequencing for detection of alternative splicing/transcripts or RNA isoforms; and (iv) direct sequencing of epigenetic/methylation markers. The direct detection of epigenetic modifications or RNA molecules can remove the need for reverse transcription for RNA sequencing and for bisulfite treatment to decipher methylation. Of note, a strong limitation arising from long reads and their analysis is the computational requirements. During the process of genome assembly, as the number of reads increases, an exponential increase is seen for the number of overlaps computed between the reads. For these reasons, many studies have demonstrated that even with the long reads technologies that have been developed, the relevance of short reads has not been lost yet. Indeed, the long reads approach is not yet used in the diagnosis of HAs.

## Application of Genetics and Genomics to Differential Diagnosis of Hereditary Anemias: Clinical and Therapeutic Implications

Although the diagnostic workflow for HAs is part of normal clinical practice, it is often very difficult to obtain differential diagnosis and classification. Indeed, there is a wide range of unspecific phenotypes and overlapping phenotypes in patients with different genetic backgrounds. For instance, CDAI/CDAII and DHS share several clinical aspects (such as low reticulocyte counts, and dyserythropoietic features of the bone marrow) and are thus often misdiagnosed, as also for CDAII and hereditary spherocytosis (Andolfo et al., 2018a). Such incorrect diagnosis can critically impact on the follow-up and therapy of these patients. Splenectomy is the most effective surgical treatment for hereditary spherocytosis, although it might not be necessary for a patient with CDAII, or even worst, it can be contraindicated in DHS because of the risk of severe thrombotic events (Stewart et al., 1996; Iolascon et al., 2017; Andolfo et al., 2018a; Picard et al., 2019).

Next-generation sequencing-based diagnosis has resulted in the modification of initial clinical diagnoses for 10–40% of patients investigated (Iolascon et al., 2020). Moreover, a recent study showed that 45.5% of patients with CDAs had conclusive diagnosis of chronic anemia arising from enzymatic defects, mainly in terms of pyruvate kinase deficiency (Russo et al., 2018). Of note, most of these cases were transfusion-dependent patients, where a classical laboratory approach (e.g., pyruvate kinase enzyme assay) was not performed or was not available. A few years ago, it was demonstrated that an allosteric activator of wild-type and mutant pyruvate kinases, AG-348 (also known as mitapivat) can increase enzymatic activities in the RBCs of these patients, and thus it has been proposed as a novel therapeutic approach for this disease (Kung et al., 2017). Indeed, a phase 2 study of patients with pyruvate kinase deficiency treated with mitapivat was recently completed (ClinicalTrials.gov: NCT02476916), which indicated that the correct identification of these patients might be valuable for guiding their treatment (Grace et al., 2019b). Co-inheritance of multiple conditions or multiple disease-associated variants are further issues that increase the complex scenario of the diagnosis of HAs. For instance, even though ektacytometry is the gold standard for DHS diagnosis, the co-inheritance of β-thalassemia or the splenectomy might modify the shape of the ektacytometry curve, thereby resulting in misdiagnosis (Lazarova et al., 2017; Zaninoni et al., 2018).

## Application of Genetics and Genomics to the Identification of Hidden Disease Mechanisms

In this section, we will briefly present the main advances and the future perspectives for the use of NGS technologies for identification of genetic modifier variants, non-coding regulatory variants, and genomic structural variants in the erythroid genes. Currently, this is an active and interesting field of research, but it is still far from application to routine clinical diagnostics.

### Polygenic Conditions and Genetic Modifiers

Identification of new causative genes has provided improved knowledge of disease etiology; however, there remains the need for better understanding of the genetic factors that can modify HA disease severity. Even diseases that are simple to diagnose can show clinical variability, and it might be that this variability itself involves genetic factors, as so-called “modifier genes” (Genin et al., 2008). One of the major advantages of the NGS approach is the identification of both the polygenic conditions and the modifier variants associated with causative mutations. Although there is no unique definition, we would define such a modifier as a gene that changes the expression of another gene at a different locus that affects the phenotypic expression of another gene, or that can modify the phenotypic manifestation of a mutant gene while not showing any effects on the normal condition (Genin et al., 2008).

In a recent study, an analysis of patients with CDAII that used a target panel of 81 genes for modifier genes identified modifier variants that could explain the clinical variability of these patients, and even among patients who shared the same pathogenic variants. Among these variants, an *ERFE* gene recurrent low-frequency variant, A260S, was shown in 12.5% of patients with CDAII who had severe phenotypes. *ERFE* encodes erythroferrone, which is a soluble protein that is secreted by erythroid precursors and that suppresses expression of hepcidin. Increased levels of ERFE are associated with the ERFE-A260S variant, which results in large impairment of the regulation pathways for iron at the level of the liver. ERFE-A260S functional characterization in a hepatic cell system showed that it has a modifier role in iron overload through the BMP/SMAD pathway. This was the first description of ERFE polymorphism as a genetic modifier variant (Andolfo et al., 2019).

Next-generation sequencing technologies allowed the identification of co-inheritance of multiple disease genotypes in a patient with DHS1 who had severe iron overload that was caused by bi-allelic mutations in *SEC23B* that had been co-inherited with a *PIEZO1* mutation (Russo et al., 2018). *PIEZO1* is a highly polymorphic gene that shows high tolerance to variations. Recently, we provided evidence that the *de novo* R1864H mutation in the *PIEZO1* gene has a phenotype modifier role that co-segregates with the inherited E2492_L2493dup, where the severe phenotype was seen for a proband with DHS1. Of note, the effects that are mediated by R1864H are mainly evident in modulation of the RBC hydration status. Indeed, we were able to show that this rare missense variant resulted in augmented K^+^ efflux when it was co-inherited with the duplication, which led in turn to the disease taking on a more severe clinical presentation (Andolfo et al., 2018c).

A recent study on 73 Asian families in an investigated that used NGS-based diagnostic approaches demonstrated that co-inherited G6PD deficiency was seen for 15% of patients with hereditary spherocytosis. *G6PD* variants worsen the phenotype by increasing the transfusion rate. Additionally, *UGT1A1* promoter variant homozygosity (Gilbert syndrome) was shown to lead to a significantly greater mean bilirubin levels, with high frequency of cholelithiasis (30% of the patients with hereditary spherocytosis analyzed) (Aggarwal et al., 2020). Hence, these new genetic technologies have provided useful tools to fill some of the gaps in our understanding of the genetic factors that modify HA disease severity.

### Non-coding Genetic Variants: Transcription Factor Binding Sites and *Cis*-Regulatory Elements

**FIGURE 2:**
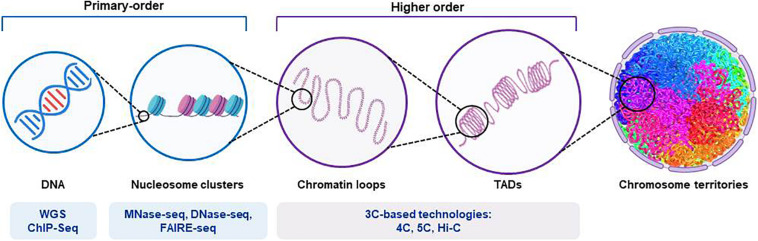
Representation of the structure of chromosomal DNA and the technical procedures for the assessment of chromatin hierarchy. The diagram shows the main features of the organization of chromatin, from those of higher order to those of primary order. The different next-generation-sequencing-based techniques used for each order degree are shown. Whole-genome sequencing (WGS) applications allow identification of both coding and non-coding regulatory variants. Chromatin immunoprecipitation sequencing (ChIP-seq) provides identification of binding sites of DNA-associated proteins, which can be used to map the global binding sites for any given protein (e.g., transcription factors). Endo-exonuclease sequencing (MNase-seq) allows identification of closed chromatin by using an endo-exonuclease to cleave the linker DNA between nucleosomes. DNase I endonuclease sequencing (DNase-seq) identifies open chromatin regions, as accessible regions of the genome are hypersensitive to this activity. Sequencing through formaldehyde-assisted isolation of regulatory elements (FAIRE-seq) is a similar method that identifies open regions of the genome. The chromosome conformation capture (3C)-based technologies (e.g., 4C, 5C, and their variants [Hi-C]) incorporate next-generation sequencing and can provide quantitative measures for intra (*cis*)-chromosomal and inter (*trans*)-chromosomal interactions. TADs, topologically associated domains.

Increased knowledge of how transcription factors and *cis*-regulatory elements influence and guide the fine-tuned processes of erythroid differentiation is critical for translation of these findings from research to possible future diagnosis and treatment of HAs. Although both targeted NGS and WES are currently used in the diagnostic workflow for these disorders, WGS might be useful in the research field to identify the presence of putative non-coding causative mutations (Figure 2). Indeed, the mutational disruption of transcription factor occupancy sites has been shown to be a pathogenic mechanism in several hematological disorders. For instance, mutations of the GATA1-binding *cis*-element in an intron of the *ALAS2* gene account for X-linked sideroblastic anemia (Kaneko et al., 2014). Similarly, disruption of the consensus binding motif for GATA1 in the promoter of *PKLR* results in severe pyruvate kinase deficiency (Manco et al., 2000; van Wijk and van Solinge, 2005).

As well as non-coding causative mutations, single nucleotide polymorphisms within *cis*-elements might also result in significant inter-individual differences in hematological parameters, and might influence therapeutic strategies. Genome-wide association studies have identified motif-disrupting single nucleotide polymorphisms in the enhancer of *BCL11A*, a critical repressor of fetal Hb levels that is associated with decreased *BCL11A* expression and elevated fetal Hb (Bauer et al., 2013). Indeed, they proposed the CRISPR–Cas9-mediated *BCL11A* enhancer editing approach in hematopoietic stem cells, as a practicable therapeutic strategy to produce durable fetal Hb in patients with β-hemoglobinopathies (Wu et al., 2019). Similarly, a polymorphism in the 5’ upstream region of *GATA1* was described as a genetic modifier in patients with CDAII (Russo et al., 2017).

Along with time-dependent regulation of erythroid gene expression, several studies have described the fine regulation that underlies tissue specificity. Thirty-five percent of genes expressed in erythroid cells have many distinct alternative promoters and first exons (Tan et al., 2006) that regulate tissue-specific isoform selection. For erythrocytes, a high number of cytoskeletal proteins and transcription factors have alternative first exons that can be upstream, positioned close together (e.g., *NRF1*) or tens of kilobases apart (e.g., *EPB4.1*), or downstream of one or more internal coding exons (e.g., *ANK1*). Of note, a 2-kb upstream region of the *ANK1* erythroid promoter leads to selective activation of its transcription in erythroid cells, while this is silent in cell types where alternative tissue-specific *ANK1* promoters are active (Gallagher et al., 2010). This sheds light on the possible pathogenic roles of mutations that do not alter coding sequences, but reduce and alter the transcription of specific isoforms (Yocum et al., 2012).

### Genomic Structural Variants: Topologically Associating Domains and Chromatin Occupancy

The assembly of DNA and proteins in chromatin is compact and organized, with the three-dimensional structure showing intricate folding. The chromosomal DNA structure is defined as ‘higher order’ and ‘primary order’ based on this folding complexity (Chang et al., 2018). The human genome is characterized by architectural features that are conserved, which include chromatin loops and domains, and nuclear bodies. It also shows non-random patterns, as seen for the location of genes and chromosomes in three-dimensional space (Misteli, 2020). With the advances seen for high-throughput sequencing, profiling of chromatin structures on a genome-wide basis has been made possible. Figure 2 briefly summarizes the main NGS-based application for the assessment of chromatin hierarchy. Generally, intra-chromosomal interactions are within regions that are known as megabase-sized topologically associating domains (TADs). These TADs have been identified as large-scale (>100 kb), highly self-interacting regions that are in spatial proximity, and that constrain the spread of heterochromatin within the same region. CCCTC-binding factor (CTCF) is enriched at the TAD boundaries, and this transcriptional repressor or insulator prevents communication between different TADs (Dixon et al., 2012). The so-called ‘loop extrusion mechanism’ that is mediated by binding of cohesin to sites on CTCF was proposed as the formation process for TADs (Fudenberg et al., 2016; Oudelaar et al., 2018; Vian et al., 2018). It is now established that disruption of the TAD boundaries is a recurrent disease mechanism that promotes aberrant gene expression due to exposure of genes to inappropriate regulatory elements (Lupianez et al., 2015; Gonzalez-Sandoval and Gasser, 2016; Sikorska and Sexton, 2020).

Genomic structural variants have been extensively studied for the globin *loci*. Hay and colleagues characterized the mouse α-globin super-enhancer, which contains five enhancer-like elements flanked by two pairs of CTCF binding sites, which define a single TAD (Hay et al., 2016).

Although TADs are strongly conserved between species, a systematic comparative analysis of chromatin occupancy of master regulators between mouse and human was performed, in the transcriptional landscape of erythroid differentiation. It was shown that transcription factor occupancy sites are well conserved across proerythroblasts and cell lines of the same species (e.g., human proerythroblasts *versus* human K562 cells) but less across different species, according to previous observations regarding the differences in timing and expression levels of some constituent erythroid genes (Pishesha et al., 2014). Conversely, substantial divergence across the mouse and human epigenomes have been reported, as demonstrated for *SEC23A* (paralog of *SEC23B*) transcription factor occupancy and histone state. Human *SEC23A* and the surrounding region are in a general state of heterochromatin, whereas the mouse *sec23a* region is open for transcription. This finding provides evidence for why hematopoietic deficiency for *SEC23B* in mice does not result in anemia or other CDAII characteristics, while it does in humans (Khoriaty et al., 2014). Moreover, the SEC23B deficient phenotype in mice can be completely rescued by expression of SEC23A from the endogenous *Sec23b* locus, which indicates that *SEC23A* and *SEC23B* have functional overlap (Khoriaty et al., 2018). In agreement with this overlap in erythroid cells, a report has shown a milder phenotype in two CDAII patients with higher SEC23A levels compared to one CDAII patient with lower SEC23A levels (Russo et al., 2013). These studies also emphasize the importance of looking at differences in chromatin occupancy between species during the development of a disease model (Ulirsch et al., 2014).

## Where Next for Genetics and Genomics in Hereditary Anemias?

The advent of high-throughput sequencing techniques has provided increased knowledge of the genetic and genomic differences that can be found among individuals. This has gradually led to changes in clinical management and therapeutic plans, which have moved from population-based approaches to providing personalized therapies for individual patients. Genetic approaches now form a routine part of studies of HAs, and they are becoming widespread in clinical practice. On the other hand, the study of non-coding genetic variants and genomic structural variants is very interesting and promising in the search for new pathogenetic mechanisms, but these remain far from being applied in clinical diagnosis.

Testing based on NGS can now provide time-effective diagnosis, while also identifying polygenic conditions and modifier variants. With our increasing knowledge of the genetic features combined with detailed phenotyping of patients with HA, this will facilitate the diagnosis of these patients, to improve their personalized clinical management, while also generating advanced telemedicine tools (Tornador et al., 2019). Third-generation sequencing still represents a revolution for NGS technologies. In terms of HAs, these approaches are still far from diagnostic applications, but they provide a valid approach for the discovery of new pathogenic mechanisms.

In the near future, this transition to personalized medicine provided by the use of genomics for HAs, as in other fields of medicine, should result in the use of individualized treatments through the targeting of the right medication to the right person at the right time, based on their unique profile.

## Author Contributions

RR, IA, RM, and BR reviewed the literature data and wrote the manuscript. AI revised the manuscript. RR designed the figures. All authors contributed to the article and approved the submitted version.

## Conflict of Interest

The authors declare that the research was conducted in the absence of any commercial or financial relationships that could be construed as a potential conflict of interest.

## Footnotes

1 www.pacb.com

2 www.nanoporetech.com
